# Roles and Mechanisms of Interleukin-12 Family Members in Cardiovascular Diseases: Opportunities and Challenges

**DOI:** 10.3389/fphar.2020.00129

**Published:** 2020-03-04

**Authors:** Jing Ye, Yuan Wang, Zhen Wang, Ling Liu, Zicong Yang, Menglong Wang, Yao Xu, Di Ye, Jishou Zhang, Yingzhong Lin, Qingwei Ji, Jun Wan

**Affiliations:** ^1^ Hubei Key Laboratory of Cardiology, Department of Cardiology, Renmin Hospital of Wuhan University, Cardiovascular Research Institute, Wuhan University, Wuhan, China; ^2^ Department of Thyroid Breast Surgery, Renmin Hospital of Wuhan University, Wuhan, China; ^3^ Department of Cardiology, the People's Hospital of Guangxi Zhuang Autonomous Region, Nanning, China

**Keywords:** cardiovascular diseases, IL-12 family members, atherosclerosis, coronary artery disease, hypertension, aortic dissection, viral myocarditis

## Abstract

Cardiovascular diseases represent a complex group of clinical syndromes caused by a variety of interacting pathological factors. They include the most extensive disease population and rank first in all-cause mortality worldwide. Accumulating evidence demonstrates that cytokines play critical roles in the presence and development of cardiovascular diseases. Interleukin-12 family members, including IL-12, IL-23, IL-27 and IL-35, are a class of cytokines that regulate a variety of biological effects; they are closely related to the progression of various cardiovascular diseases, including atherosclerosis, hypertension, aortic dissection, cardiac hypertrophy, myocardial infarction, and acute cardiac injury. This paper mainly discusses the role of IL-12 family members in cardiovascular diseases, and the molecular and cellular mechanisms potentially involved in their action in order to identify possible intervention targets for the prevention and clinical treatment of cardiovascular diseases.

## Introduction

To date, cardiovascular disease remains the leading killer worldwide, especially in less-developed areas (Leong et al., 2017). It is not only a serious threat to patients' lives, but also poses a serious psychological burden to patients and their families. Although a large number of useful drugs and new technologies have been widely used in clinical treatment over recent years and have significantly improved survival rates, the overall prognosis of cardiovascular diseases is still very poor, and the death rate related to cardiovascular diseases is far higher than that of other diseases, even malignant tumors (Donofrio et al., 2014; Dukkipati et al., 2017; Bethel et al., 2018).

There are four members of the interleukin-12 (IL-12) family, including IL-12, IL-23, IL-27, and IL-35. An obvious feature of IL-12 family members is that each consists of two heterogeneous dimers, including an α subunit (p19, p28, and p35) and a β subunit [p40 and Epstein-Barr virus-induced protein 3 (EBI3)] (Vignali and Kuchroo, 2012; Sun et al., 2015). Therefore, deletion of either an α or β subunit can cancel the biological effects of the IL-12 family cytokines. Interestingly, the receptor for IL-12 family members also consists of two protein chains. Among them, the IL-12 receptor (IL-12R) utilizes IL-12Rβ1 and IL-12Rβ2, IL-23 signaling employs IL-12Rβ1 and IL-23R, and IL-27 signals through gp130 and WSX-1; however, an exception is IL-35R, which consists of two downstream signals, including gp130-gp130 or IL-12Rβ1-IL-12Rβ1 (Presky et al., 1996; Oppmann et al., 2000; Pflanz et al., 2004; Collison et al., 2012). Molecular signaling mechanisms involving IL-12 family members are basically similar, and are all mediated by members of the Janus kinase (JAK) signal transducers and activators of transcription (STAT) family, especially JAK1/2-STAT1/3/4 (Ihle, 1995; O'Shea et al., 2002; Delgoffe et al., 2011). All IL-12 family members can be secreted by both immune and non-immune cells (Vignali and Kuchroo, 2012; Sun et al., 2015). For the immune cells, IL-12, IL-23, and IL-27 are mainly derived from effector T lymphocytes, macrophages, and dendritic cells, while IL-35 is mainly secreted by T helper cells (Tregs) (Langrish et al., 2004; Hunter, 2005; Collison et al., 2009; Collison et al., 2010; Andrews et al., 2016; Wei et al., 2017). IL-12 and IL-23 are considered to be pro-inflammatory factors that amplify downstream inflammatory signals. IL-35 plays an anti-inflammatory role and protects against tissue damage mediated by inflammatory responses, while IL-27 has a two-sided effect on the regulation of inflammation, in which it can not only play an anti-inflammatory role, but also a pro-inflammatory role, depending on the inflammatory environment (Ma and Trinchieri, 2001; Kastelein et al., 2007; Collison and Vignali, 2008; Vignali et al., 2008; Cox et al., 2011; Vignali and Kuchroo, 2012; Wojno and Hunter, 2012; Sun et al., 2015). The subunits, receptors, signaling pathways, and regulatory roles of the members of the IL-12 family in inflammation are listed in **Table 1**.

**Table 1:** The subunits, receptors, signaling pathways of the IL-12 family members.

	IL-12	IL-23	IL-27	IL-35	References
Subunits	α: p35	α: p19	α: p28	α: p35	Vignali and Kuchroo, 2012; Sun et al., 2015
	β: p40	β: p40	β: EBI3	β: EBI3	
Receptors	IL-12Rβ1+IL12-Rβ2	IL-12Rβ1+IL-23R	gp130+ WSX-1	1. gp130+IL-12Rβ2 2. gp130+gp130 3. IL-12Rβ2+lL-12Rβ2	Presky et al., 1996; Oppmann et al., 2000; Pflanz et al., 2004; Vignali and Kuchroo, 2012; Collison et al., 2012; Sun et al., 2015
Pathways	JAKs: JAK2, TYK2 STATs: STAT4	JAKs: JAK2, TYK2 STATs: STAT3, STAT4	JAKs: JAK1, JAK2 STATs: STAT1, STAT3	JAKs: JAK1, JAK2 STATs: STAT1, STAT3, STAT4	Ihle, 1995; O'Shea et al., 2002; Delgoffe et al., 2011; Vignali and Kuchroo, 2012; Sun et al., 2015
Main sources	Mø, Th1	Mø, activated DCs,	Myeloid cells, such as Mø and activated DCs	Treg	Langrish et al., 2004; Hunter, 2005; Collison et al., 2009; Collison et al., 2010; Vignali and Kuchroo, 2012; Sun et al., 2015; Andrews et al., 2016; Wei et al., 2017
Other sources	activated DCs, NK, B cells, Th9, Th17	γδ T cells, B cells, NK cells, ECs, innate lymphoid cells	T cells, B cells, epithelial cells, plasma cells, and ECs	Activated DCs, Mø, placental trophoblast cells	Langrish et al., 2004; Hunter, 2005; Collison et al., 2009; Collison et al., 2010; Vignali and Kuchroo, 2012; Sun et al., 2015; Andrews et al., 2016; Wei et al., 2017
Role in immune response	Induce Th1 and Mø1 differentiation	Induce and promote th17 differentiation	IL-27 alone has no apparent stimulatory properties, collaboration with other ILs promote or inhibit T cell differentiation and proliferation	Promote Treg activity, suppress the Teff cell (Th1, and Th17 ) activity	Ma and Trinchieri, 2001; Kastelein et al., 2007; Collison and Vignali, 2008; Vignali et al., 2008; Cox et al., 2011; Vignali and Kuchroo, 2012; Wojno and Hunter, 2012; Sun et al., 2015
Regulation of inflammation	Except inflammatory environment induced by DOX or Ang II, all play pro-inflammatory role	Always play a pro-inflammatory roles, no anti-inflammatory effects had been reported	Not only play an anti-inflammatory role, but also play a pro-inflammatory effects, may be associated with inflammatory microenvironment	Always relieves the inflammatory response	Davenport and Tipping, 2003; Vignali and Kuchroo, 2012; Jin et al., 2012; Koltsova et al., 2012; Li et al., 2012; Yan et al., 2012; Jääskeläinen et al., 2013; Abbas et al., 2015; Subramanian et al., 2015; Sun et al., 2015; Andrews et al., 2016; Tao et al., 2016; Hu et al., 2016; Gregersen et al., 2017; Fatkhullina et al., 2018; Ye et al., 2018b; Jia et al., 2019; Liu et al., 2019; Vargas-Alarcón et al., 2019; Ye et al., 2019

Mø, macrophages, Mø1, M1 macrophages; DCs, dendritic cells; natural killer cell; endothelial cells; DOX; doxorubicin.

## Interleukin-12 Family Members and Cardiovascular Disease

### Interleukin-12 Family Members and Atherosclerosis, Coronary Artery Disease

Atherosclerosis and coronary artery diseases due to atherosclerosis are chronic inflammatory disorders, and infiltration by immune cells and inflammatory factors can be observed at all stages of disease development (Peter et al., 2009; Longenecker et al., 2016; Rahman and Fisher., 2018). IL-12 family members have significantly higher levels of expression in patients with atherosclerosis and coronary artery disease, and are closely related to the progression of these diseases.

### Clinical Data

Previous studies reported that plasma IL-12 concentrations are significantly increased in many types of atherosclerosis and atherosclerotic cardiovascular disease, including stable angina pectoris (SAP), non-ST segment elevation myocardial infarction (NSTEMI), ST-elevation myocardial infarction (STEMI), acute myocardial infarction (AMI), and gradually increased SAP, unstable angina pectoris (UAP), and AMI (Zhou et al., 2001; Correia et al., 2010; Lin et al., 2012; Yong et al., 2013; Chistiakov et al., 2015; Opstad et al., 2016; Zykov et al., 2016). Clinical data showed that patients with coronary artery disease exhibit higher circulating IL-23 levels (Lin et al., 2012; Abbas et al., 2015; Sun et al., 2019a). In coronary artery disease patients who underwent percutaneous coronary intervention (PCI) with drug-eluting stents (DES), subjects with in-stent restenosis show higher circulating IL-23 levels in peripheral blood mononuclear cells (PBMCs) (Khojasteh-Fard et al., 2012). Numerous studies have confirmed that IL-27 expression is increased in plasma and plaques in the coronary and carotid arteries of coronary artery disease patients (Kempe et al., 2009; Jin et al., 2012; Lin et al., 2012; A Shahi et al., 2015; Gregersen et al., 2017). Abundant evidence identifies that IL-35 expression is significantly reduced in patients with coronary artery disease; plasma IL-35 levels are gradually reduced in SAP, UAP, and AMI patients, and decreased plasma IL-35 levels are inversely correlated with the left ventricular ejection fraction (LVEF) in coronary artery diseases (Lin et al., 2012; Rasa et al., 2018; Zhu et al., 2018).

Gene polymorphisms in members of the IL-12 family have been reported to be associated with the occurrence or progression of coronary artery disease. An IL-23R polymorphism was observed to be related to coronary artery disease, and the IL-23R rs6682925T/C polymorphism may independently relate to the presence of coronary artery disease (Zhang et al., 2014a). *IL-27* gene polymorphism had no effect on the presence of subclinical atherosclerosis, while closely related to atherosclerosis and coronary artery disease, rs26528 T and rs40837 A alleles significantly reduced the risk of coronary artery disease (Posadas-Sánchez et al., 2017; Vargas-Alarcón et al., 2019). No research about gene polymorphisms of IL-12 and IL-35 and the presence of coronary artery disease was reported yet.

### Animal Studies

Elevated serum IL-12 levels are observed of atherosclerosis in ApoE-KO mice, and increased IL-12 levels are associated with the progression of atherosclerosis (Jääskeläinen et al., 2013). Accumulating animal study reports also demonstrate that treatment with exogenous recombinant murine IL-12 significantly aggravates the progression of atherosclerosis, and increases aortic atherosclerotic plaque areas in both ApoE-knockout mice and in low density lipoprotein (LDL) receptor-deficient mice, while cancelation the biological effects of IL-12 can significantly diminish such effects (Lee et al., 1999; Davenport and Tipping, 2003; Hauer et al., 2005). In a murine myocardial infarction model, canceling the biological effects of IL-12 alleviates cardiac dysfunction by promoting angiogenesis (Kan et al., 2016). In a recent study, Shi et al. reported that knockout of IL-12p35 subunit, which can cancel the biological effects of IL-12 and IL-35, significantly aggravated Th1/Th2 and Th17/Treg imbalance and increased atherosclerotic plaque areas in ApoE mice, which may suggest that the pro-atherosclerotic effects of IL-12 can be mediated by promoting the CD4+ T lymphocyte differentiation imbalance (Huang et al., 2019).

The role of IL-23 in atherosclerosis is controversial. Therapy involving IL-23p19, a subunit of IL-23, had no significant effect on atherosclerosis development in ApoE-deficient mice, although inflammatory responses were reduced (Wang et al., 2019). Another study reported that there was no significant difference in atherosclerotic area between low-density lipoprotein receptor (LDLR) knockout mice and IL-23 + LDLR double-knockout mice, after they were all fed with high-fat diet (Engelbertsen et al., 2018). A recent study reported that deficiency of IL-23 significantly decreased IL-22 expression in ApoE-knockout mice, and also reduced expression of IL-22, thereby relieving the release of inflammatory substances, and thus alleviating the process of atherosclerosis (Fatkhullina et al., 2018). Subramanian et al. reported that granulocyte-macrophage colony stimulating factor (GM-CSF) up-regulates the expression of IL-23, which further promotes the differentiation of macrophages and atherosclerosis development (Subramanian et al., 2015). These studies suggest that IL-23 has a strong regulatory effect on inflammation mediated by a high-fat diet in both ApoE-knockout mice and LDL-R-knockout mice (Subramanian et al., 2015; Engelbertsen et al., 2018; Fatkhullina et al., 2018; Wang et al., 2019), while the special role of IL-23 in atherosclerotic progression is unclear and further studies are needed to clarify this aspect.

Both the effects of IL-27R and IL-27 on atherosclerosis were reported. Koltsova et al. found that knockout of IL-27R significantly enhanced Th17 immune responses, up-regulated inflammatory responses, promoted the expression of tumor necrosis factor (TNF) and IL-17A, and further promoted the development of atherosclerosis in ApoE-deficient mice (Koltsova et al., 2012). Hirase et al. also reported that knockout of IL-27 plays similar roles in atherosclerosis development in LDLR-knockout mice; the mechanism may be related to the promotion of macrophage differentiation (Hirase et al., 2013). Ryu et al. found that in a high-fat diet-treated ApoE-knockout mouse atherosclerotic model, blockade of IL-27 signaling increased the plaque area *via* promotion of autoimmune follicular helper T cell responses (Ryu et al., 2018). These results suggest that IL-27 may be an important target for the treatment and prevention of atherosclerosis and coronary artery disease by inhibiting the differentiation of various immune cells and reducing inflammatory responses, thereby alleviating atherosclerotic progression. Hence, IL-27 may be an important target for the treatment and prevention of atherosclerosis and coronary artery disease.

Contrary to clinical experiments, as an anti-inflammatory cytokine, IL-35 expression in mouse atherosclerotic plaques was significantly increased (Wang et al., 2014). A small number of other studies, however, have reported increased IL-35 expression in atherosclerotic plaques and serum in ApoE mice fed with a high-fat diet, as well as in the plasma of patients with coronary artery disease (Gorzelak-Pabiś et al., 2017; Li et al., 2018). Using a mouse model of atherosclerosis, administration of recombinant mouse IL-35 significantly decreased plaque area in the aortic root, and Treg immune responses were also found to be enhanced (Tao et al., 2016). In a recent study, Shi et al. reported that knockout of IL-12p35 subunit, which can cancel the biological effects of IL-12 and IL-35, significantly aggravated Th1/Th2 and Th17/Treg imbalance and increased atherosclerotic plaque areas in ApoE mice, given that mouse IL-35 reverses Th35/Treg imbalance and up-regulates atherosclerosis development, whereas there were no effects on Th1/Th2 imbalance (Huang et al., 2019). In a recently published study, two subunits of IL-35 found in the left anterior descending branch following its ligation induced myocardial infarction in the heart tissues in mice. In addition, exogenous IL-35 treatment can significantly reduce infarct area of the left ventricle and reduce the incidence of left ventricular rupture; the mechanisms underlying this phenomenon may be related to the inhibitory role of IL-35 in the apoptosis of myocardial macrophages, thus increasing the differentiation of M2 macrophages and augmenting the expression of collagen (Jia et al., 2019). In rat models of coronary artery disease, IL-35 treatment significantly promotes early drug-eluting stent endothelialization; its mechanism may be related to the regulation of the activation of M2 macrophages (Liu et al., 2019). These studies have demonstrated that IL-35 regulates the differentiation of various immune cells involved in the progression of atherosclerotic heart disease.

### Interleukin-12 Family Members and Hypertension

Hypertension is a complex group of clinical syndromes. Although the specific mechanisms remain unclear, it has been demonstrated that a variety of pathological factors are involved in the process of hypertension, among which immune responses and inflammation are most closely related to hypertension (Kirabo et al., 2014; Pober, 2014; Guzik and Touyz, 2017).

So far, little research has been conducted on IL-12 family members and hypertension. Data from clinical experiments reported that plasma IL-12 levels are significantly increased in hypertensive patients, and are positively correlated with both systolic blood pressure (SBP) and diastolic blood pressure (DBP) (Ye et al., 2019). IL-12 polymorphism is closely related to the incidence of hypertension-induced complications: hypertension patients who carry the *IL12B* 1159 A/A genotype exhibit a lower risk of incidence of stroke, while *IL12B* A/A carriers have an elevated risk of stroke (Timasheva et al., 2008).

In an animal study, angiotensin II (Ang II) infusion significantly increased aortic IL-12p35 expression and macrophages were the primary source (Ye et al., 2019). In an Ang II-induced mouse hypertension model, IL-12p35 knockout promoted M1 macrophage differentiation and elevated blood pressure, while IL-12 treatment unexpectedly lowered blood pressure (Ye et al., 2019). Another study reported that knockdown of IL-12p35 did not affect Ang II-induced hypertension (Li et al., 2012). One possible reason for this is that the IL-12p35 knockout mice in that study were treated for only a week, which is too short a period for blood pressure to change. Little research has been conducted regarding IL-23, IL-27, and IL-35 in relation to hypertension. One group reported that in deoxycorticosterone acetate and Ang II-treated mice, deficiency of IL-17 could decrease IL-23 expression and accelerate kidney injury (Krebs et al., 2014), and another study found that treatment with recombinant mouse IL-35 had no effects on blood pressure in Ang II-treated mice (Ye et al., 2019).

### Interleukin-12 Family Members and Aortic Aneurysms and Aortic Dissection

Aortic aneurysms and aortic dissection are both degenerative lesions of the aorta and share the same pathological mechanisms, such as the excessive loss of aortic extracellular matrix mediated by multiple pathological factors, especially local aortic inflammation (Mallat et al., 2016; Rabkin, 2017; Raffort et al., 2017; Sherifova and Holzapfel, 2019).

There have been few studies regarding IL-12 family members, aortic aneurysms, and aortic dissection. Davis et al. reported that IL-12 levels in aortic tissue and serum were not significantly different in patients with abdominal aortic aneurysms compared to those in patients without abdominal aortic aneurysms (Davis et al., 2001). In aortic dissection patients, decreased plasma IL-35 concentrations were observed compared to non-aortic dissection patients (Ye et al., 2018a). In addition, no studies have been conducted on IL-12 family members and aortic aneurysms and aortic dissection.

Only one recent animal study reported that deletion of IL-27R reduced the formation of abdominal aortic aneurysm in ApoE deficiency mice, the mechanisms may be associated with a blunted accumulation of myeloid cells in the aorta (Peshkova et al., 2019).

### Interleukin-12 Family Members and Cardiac Fibrosis

Cardiac fibrosis is a common feature of many heart diseases and is closely related to deterioration in cardiac function. The essence of cardiac fibrosis is that pathological factors activate cardiac fibroblasts, leading to abnormal deposition and increased numbers of cardiac collagen fibers (Moore-Morris et al., 2015; Gorabi et al., 2019).

All studies on IL-12 family members and cardiac fibrosis have been focused on animal studies and no related clinical studies have been reported. In an earlier report, the authors reported that infusion with Ang II increases cardiac IL-12 expression derived from cardiac macrophages; detection of IL-12 promotes the activation of CD4+ T lymphocytes and increases differentiation of M2 macrophages, thereby up-regulating the activation of the transforming growth factor-β1 (TGF-β1) signaling pathway, which then aggravates cardiac fibrosis (Li et al., 2012). In mouse models of myocardial infarction, deletion of *IL-23* significantly reduces the expression of multiple fibrosis markers, including α-smooth muscle actin (α-SMA), collagen I, and collagen III (Savvatis et al., 2014). Unexpectedly, Yan et al. also reported that IL-23 deficiency amplifies the inflammatory response and promotes the release of various inflammatory factors, especially IL-17, which further promotes the infiltration and deposition of γδT cells in the left ventricle, promotes the apoptosis of cardiomyocytes, and aggravates cardiac fibrosis in a murine myocardial infarction model (Yan et al., 2012). In addition, IL-12p35 knockout increased the levels of cardiac mitochondrial reactive oxygen species (ROS) and calcium ion overload, which further aggravated mitochondrial dysfunction and energy failure, increased myocardial cell apoptosis, worsened cardiac dysfunction, and increased cardiac fibrosis in 25-month-old aging mice (Ye et al., 2020). However, how the cytokines IL-12 and IL-35 mediate these biological effects is currently unknown. Furthermore, no studies concerning IL-27 and IL-35 related to cardiac fibrosis have been reported.

### Interleukin-12 Family Members and Cardiac Ischemia Reperfusion Injury

Ischemia reperfusion injury of the heart is an important issue that cannot be ignored in heart transplantation. A large number of studies have confirmed that myocardial apoptosis mediated by inflammatory responses after cardiac reperfusion is one of the most important mechanisms of ischemia reperfusion injury of the heart (Shin et al., 2017; Schanze et al., 2019).

Numerous animal studies have reported that members of the IL-12 family are involved in cardiac ischemia-reperfusion injury. In a recent study, Yan found that dectin-2 deficiency could protect against cardiac ischemia-reperfusion injury *via* alleviating Th1 immune responses and further decreasing IL-12 expression (Yan et al., 2017). An earlier study reported that high-mobility group box 1 (Hmgb-1) promoted ischemia-reperfusion injury in a mouse cardiac transplantation model (Zhu et al., 2013). In subsequent studies, pentraxin-3 and necrostatin-1 were also found to attenuate ischemic reperfusion injury by decreasing the expression of IL-23 (Zhang et al., 2014b; Zhu et al., 2014). Hu et al. reported that administration of mouse anti-IL-23 neutralizing antibody significantly reduced the expression of inflammatory markers such as IL-6, tumor necrosis factor α (TNF-α), and pro-oxidant markers such as malondialdehyde (MDA), and decreased the levels of superoxide dismutase (SOD), thereby relieving cardiac ischemia reperfusion injury (Hu et al., 2016). Up-regulation of cardiac IL-23 expression by adenovirus significantly increased the expression of serum lactate dehydrogenase (LDH) and creatine kinase myocardial band (CK-MB), elevated the expression of apoptosis-related proteins and infarcted areas, and these effects could be reversed by AG490, an inhibitor of the JAK2-STAT pathway (Liao et al., 2017). However, the roles of both IL-27 and IL-35 in cardiac ischemia and reperfusion injury have not been studied to date.

### Interleukin-12 Family Members and Atrial Fibrillation

Atrial fibrillation is one of the most common arrhythmias and can lead to vascular embolizations, the most serious of which is cerebral artery embolization. Literature reports confirm that the mechanism of atrial fibrillation may be closely related to the occurrence of atrial fibrosis (Jalife and Kaur, 2015; Nattel, 2017).

Previous clinical studies have reported that elevated IL-12 expression is observed in left atrial tissues of atrial fibrillation patients (Stein et al., 2008; Lappegård et al., 2013). Chen et al. found that *IL-27* genetic variants, including the rs153109 G allele and GG genotype, increased the occurrence of atrial fibrillation in the Chinese Han population (Chen et al., 2017a).

Recent animal studies have reported that inhibition of Ang II-induced M1 macrophage differentiation and reduction of IL-12 release can reduce the occurrence of atrial fibrosis and atrial fibrillation (Sun et al., 2019b). No studies on the expression and mechanisms of the involvement of IL-23 and IL-35 in atrial fibrillation have been reported.

### Interleukin-12 Family Members and Viral Myocarditis

Viral myocarditis is an uncommon heart disease. The death rate involving severe myocarditis exceeds that of AMI. Immune responses induced by viral infection are an important cause of myocardial injury in viral myocarditis (Chen et al., 2013; Pollack et al., 2015).

So far, although no clinical experiments have been reported on IL-12 family members and viral myocarditis, a large number of animal experiments have confirmed that all IL-12 family members are associated with viral myocarditis. Substantial evidence indicates that IL-12 expression is increased in both plasma and heart tissue of coxsackievirus B3-induced viral myocarditis in mice. In addition, elevated IL-12R levels were also found in heart tissue of mice with viral myocarditis (Fairweather et al., 2003; Nyland et al., 2012; Jenke et al., 2014; Zha et al., 2015; Miteva et al., 2017; Zhang et al., 2017). In an earlier study, the authors found that treatment with coxsackievirus B3 significantly increased both cardiac IL-12p35 and IL-12p40 expression, and treatment with recombinant mouse IL-12 and anti-IL-12 neutralizing antibodies reduced and increased mortality, respectively, in mice with viral myocarditis (Shioi et al., 1997). In a subsequent study, Nishio et al. reported that carvedilol treatment increases both IL-12 and interferon-γ (IFN-γ) expression, thereby reducing virus replication and thus improving survival rates in viral myocarditis mice (Nishio et al., 2003). In another study, Fairweather et al. demonstrated that the protective effect of IL-12 in viral myocarditis is mediated by activation of the STAT4 pathway and promotion of IFN-γ release. Knockout of the STAT4 pathway and IFN-γ can significantly reverse the protective effects of IL-12 and aggravate myocardial cell injury and mortality (Fairweather et al., 2005). Similarly, circulating IL-23 levels were also observed to be increased in coxsackievirus B3-induced mouse viral myocarditis (Yang et al., 2011; Sesti-Costa et al., 2017). Although there are no direct reports concerning the effects of IL-23 on viral myocarditis, emodin can reduce myocardial injury and mortality mediated by viral myocarditis by reducing the expression of IL-23, indicating that IL-23 can aggravate myocardial injury in viral myocarditis (Jiang et al., 2014). In an initial study, Kong et al. found that IL-27 levels were elevated in mice with viral myocarditis, and regulated IL-17 expression, suggesting that IL-27 may be involved in the development of viral myocarditis (Kong et al., 2014). In a follow-up study, Zhu et al. found that IL-27 inhibited immune responses to Th17 and reduced the expression of IL-17, thereby protecting against coxsackievirus B3-induced viral myocarditis (Zhu et al., 2015). Unlike other members of the IL-12 family, IL-35 levels were found to be reduced in a mouse model of viral myocarditis, and were negatively correlated with the severity of viral myocarditis, as was the frequency of Tregs (Hu et al., 2014; Ouyang et al., 2017; Xu et al., 2018). In addition, up-regulation of IL-35 expression can significantly reduce Th17-mediated immune responses, and decrease IL-17 expression, thereby alleviating cardiac injury caused by viral myocarditis (Hu et al., 2014). These studies have confirmed that all IL-12 family members are involved in the course of viral myocarditis, and the mechanisms involved in their action are related to the regulation of Th1 and Th17 immune responses. Whether other immune cells are involved needs further confirmation.

### Interleukin-12 Family Members and Cardiomyopathy

Cardiomyopathy is a rare heart disease characterized by enlarged ventricular spaces with unknown etiology. Its pathological mechanisms are very complex, and many factors, including genetic variation, can induce its occurrence (Heinig et al., 2017).

Data from previous clinical experiment reported that IL-12 expression was found to be unchanged in patients with idiopathic dilated cardiomyopathy and their relatives (Marriott et al., 1996), whereas IL-12 expression was found to be elevated in patients with autoimmune cardiomyopathy or alcoholic cardiomyopathy (Izumi et al., 2000; Jenke et al., 2013; Panchenko et al., 2015). *IL-12R* gene polymorphisms, including *IL-12B* 3' UTR C and *IL-12B* 3' UTR CC, result in significantly higher gene expression, and may increase the incidence of Chagas cardiomyopathy (Zafra et al., 2007). Similar to the IL-12 expression trends, circulating or cardiac IL-23 levels were found to be increased in patients with dilated cardiomyopathy, and with idiopathic dilated cardiomyopathy (Yi et al., 2009; Li et al., 2010; Myers et al., 2016). Individuals with *IL-12, IL-23R* polymorphisms, such as SNP rs10889677, are more susceptible to dilated cardiomyopathy among the Chinese Han population, rather than those with rs1884444 and rs11465817 (Chen et al., 2009). Elevated *IL-27* mRNA levels were observed in the heart tissue of human dilated cardiomyopathy patients, and an *IL-27* gene polymorphism involving SNP rs153109, rather than SNP rs17855750, predisposes to dilated cardiomyopathy in the Chinese Han population (Noutsias et al., 2011; Chen et al., 2017b). The expression of IL-35 in human cardiomyopathy has not been reported.

In a mouse model of cardiac myosin immunized-mice, the absence of IL-12R significantly reduced cardiac immune responses and delayed the progression of autoimmune cardiomyopathy, whereas knockout of the STAT4 pathway and IFN-γ significantly reversed the protective effect of IL-12 in autoimmune cardiomyopathy (Afanasyeva et al., 2001). In another study, Fairweather et al. found that knockout of IL-12R significantly slowed the progression of dilated cardiomyopathy in murine chronic viral myocarditis (Fairweather et al., 2004). Using IL-12p35 and IL-12p40 knockout mice and anti-IL-23 neutralizing antibodies, other researchers found that IL-23, rather than IL-12, exacerbated the progression of a localized underwear purchase response in the heart and autoimmune myocarditis, which could be blocked by anti-IL-17 neutralizing antibodies (Sonderegger et al., 2006). In a recent study, Wu et al. demonstrated that IL-23 is necessary to initiate cardiac autoimmunity by stimulating the activation and differentiation of CD4+ T lymphocytes (Wu et al., 2016). In contrast there have been no studies regarding cardiomyopathy and involvement of IL-27 and IL-35.

### Interleukin-12 Family Members and Other Cardiovascular Diseases

IL-12 family members are also implicated in other cardiovascular diseases that are less common, such as congenital heart disease, ventricular fibrillation, and rejection after cardiac transplantation.

In earlier studies, it was reported that in young children with congenital heart disease, circulating IL-12 levels did not exhibit significant change after surgery (Madhok et al., 2006). Furthermore, the *IL-27* gene polymorphism, SNP rs153109, rather than rs17855750, is associated with congenital atrial septal defects and congenital ventricular septal defects (Zhang et al., 2016). In addition, IL-12 levels in plasma and brain tissue are significantly increased in an animal model of cardiac arrest after ventricular fibrillation. (Janata et al., 2014; Heo et al., 2017). In a mouse model of heart transplantation, administration of an anti-IL-12p40 antibody significantly reduced invasion by γδT cells, reduced the expression of various inflammatory factors, and greatly improved the survival of mice (Wang et al., 2012). In an animal model of acute myocardial injury induced by the chemotherapeutic drug doxorubicin, deletion of *IL-12p35* significantly increased cardiac injury, which was associated with increased inflammatory responses, oxidative stress, apoptosis, and autophagy. Treatment with recombinant mouse IL-12 significantly reversed these effects, suggesting that both IL-12 and IL-35 may play protective roles in cardiac injury induced by doxorubicin (Jia, 2018; Ye et al., 2018b; Ye et al., 2018c).

## Conclusions

The current review sought to describe the composition, structure, molecular receptors, signaling pathways, and regulatory roles of each IL-12 family member. The expression of IL-12 family members in different cardiovascular diseases in humans and animals, and the regulatory effect of IL-12 family members on inflammatory response in different cardiovascular models are also summarized in this paper in **Tables 2** and **3**. In addition, we also described the roles and possible mechanisms of involvement of IL-12 members in different cardiovascular diseases. Among these IL-12 family members, IL-12 can aggravate a variety of cardiovascular diseases, in addition to acute cardiac injury induced by doxorubicin, and hypertension prompted by Ang II. IL-23 mostly plays a role in injury. IL-27 has a two-sided regulatory effect in cardiovascular disease, with both protective and damaging effects; while IL-35 has been found to play a protective role in all cardiovascular diseases. Just as **Table 4**. Although IL-12 family members are involved in various biological effects such as inflammatory responses, oxidative stress, and apoptosis, the regulation of immune cell differentiation and inflammation is still the most important mechanism for the involvement of IL-12 in the development of cardiovascular diseases. In view of this, IL-12 family members may be potential targets for clinical prevention, intervention, and treatment of cardiovascular diseases. Hence, when considering IL-12 family members as potential targets for cardiovascular disease therapy, the influence of other cytokines and interactions involving interleukin family members should be considered.

**Table 2:** Expression of IL-12 family members in cardiovascular diseases.

	Diseases	IL-12	IL-23	IL-27	IL-35	References
Mouse	AS	Increase	Increase	–	Contro	Jääskeläinen et al., 2013; Wang et al., 2014; Subramanian et al., 2015
	IR	Increase	Increase	–	–	Zhu et al., 2013; Zhang et al., 2014b; Zhu et al., 2014; Yan et al., 2017
	CAD	Increase	–	–	Decrease	Wang et al., 2014; Kan et al., 2016
	Hypertension	Increase	Increase	–	–	Li et al., 2012; Krebs et al., 2014; Ye et al., 2019
	Viral myocarditis	Increase	Increase	Increase	Decrease	Fairweather et al., 2003; Yang et al., 2011; Nyland et al., 2012; Jenke et al., 2014; Kong et al., 2014; Hu et al., 2014; Zha et al., 2015; Zhang et al., 2017; Miteva et al., 2017; Sesti-Costa et al., 2017; Ouyang et al., 2017; Xu et al., 2018
	CM	–	–	–	–	–
	AA, AD	–	–	–	–	–
	AF	–	–	–	–	–
Human	AS	–	–	Increase	–	Gregersen et al., 2017
	IR	–	–	–	–	–
	CAD	Increase	Increase	Increase	Contro	Zhou et al., 2001; Kempe et al., 2009; Correia et al., 2010; Lin et al., 2012; Khojasteh-Fard et al., 2012; Jin et al., 2012; Yong et al., 2013; Chistiakov et al., 2015; Abbas et al., 2015; A Shahi et al., 2015; Zykov et al., 2016; Opstad et al., 2016; Zhu et al., 2018; Rasa et al., 2018; Sun et al., 2019a
	Hypertension	Increase	–	–	–	Ye et al., 2019
	Viral myocarditis	–	–	–	–	–
	CM	Increase	Increase	Increase	–	Marriott et al., 1996; Izumi et al., 2000; Yi et al., 2009; Li et al., 2010; Jenke et al., 2013; Panchenko et al., 2015; Myers et al., 2016; Wu et al., 2016
	AA, AD	Unchanged	–	–	Decrease	Davis et al., 2001; Ye et al., 2018a
	AF	Increase	–	–	–	Stein et al., 2008; Lappegård et al., 2013

AS, atherosclerosis; IR, ischemia-reperfusion; CAD, coronary artery diseases; CM, cardiomyopathy; AA, arterial aneurysm; AD, aortic dissection; AF, atrial fibrillation; Contro, controversial.

**Table 3:** Regulation of different inflammatory environments by members of the interleukin-12 family.

Organ	Mouse	Model	IL-12	IL-23	IL-27	IL-35	References
**Heart**	Wild type	Ang II	Down	–	–	–	Li et al., 2012
	Wild type	CVB3	Both	Up	Down	–	Shioi et al., 1997; Nishio et al., 2003; Fairweather et al., 2005; Jiang et al., 2014; Zhu et al., 2015
	Wild type	LLDB	Up	–		Down	Kan et al., 2016; Jia et al., 2019; Liu et al., 2019
	Wild type	IR	Up	Up	–	–	Zhu et al., 2013; Zhang et al., 2014b; Zhu et al., 2014; Hu et al., 2016; Yan et al., 2017; Liao et al., 2017
	Wild type	DOX	Down	–	–	Down	Jia, 2018; Ye et al., 2018b; Ye et al., 2018c
**Aorta**	Wild type	Ang II	Down	–	–	–	Ye et al., 2019
	ApoE-/-, LDLR-/-	HFD	Up	Both	Both	Down	Lee et al., 1999; Davenport and Tipping, 2003; Hauer et al., 2005; Koltsova et al., 2012; Hirase et al., 2013; Subramanian et al., 2015; Kan et al., 2016; Tao et al., 2016; Engelbertsen et al., 2018; Fatkhullina et al., 2018; Ryu et al., 2018; Huang et al., 2019; Huang et al., 2019; Wang et al., 2019; Jia et al., 2019; Liu et al., 2019

Ang II, angiotensin II; CVB3, Coxsackievirus B3; LLDB, ligation of left anterior descending branch; IR, ischemia-reperfusion; DOX, doxorubicin; HFD, high-fat diet.

Up: magnify inflammatory response.

Down: alleviate inflammatory response.

Both: both the magnification and reduction of inflammatory response were reported.

**Table 4:** Regulation of IL-12 family members on cardiovascular diseases.

Diseases	IL-12	IL-23	IL-27	IL-35	References
**Atherosclerosis**	Aggravate	Contro	Aggravate	Alleviate	Lee et al., 1999; Davenport and Tipping, 2003; Hauer et al., 2005; Koltsova et al., 2012; Hirase et al., 2013; Wang et al., 2014; Subramanian et al., 2015; Tao et al., 2016; Gorzelak-Pabiś et al., 2017; Engelbertsen et al., 2018; Fatkhullina et al., 2018; Li et al., 2018; Ryu et al., 2018; Huang et al., 2019; Wang et al., 2019
**Cardiac ischemia reperfusion**	Aggravate	Aggravate	–	–	Zhu et al., 2013; Zhang et al., 2014b; Zhu et al., 2014; Hu et al., 2016; Liao et al., 2017; Yan et al., 2017
**CAD**	Aggravate	–	–	Alleviate	Kan et al., 2016; Jia et al., 2019; Liu et al., 2019
**Hypertension**	Alleviate	–	–	No effect	Li et al., 2012; Krebs et al., 2014; Ye et al., 2019
**Aortic dissection**	–	–	Aggravate	–	Peshkova et al., 2019
**Viral myocarditis**	Contro	Aggravate	Alleviate	Alleviate	Shioi et al., 1997; Nishio et al., 2003; Fairweather et al., 2005; Yang et al., 2011; Jiang et al., 2014; Kong et al., 2014; Hu et al., 2014; Zhu et al., 2015; Sesti-Costa et al., 2017; Ouyang et al., 2017; Xu et al., 2018
**Cardiomyopathy**	Aggravate	Aggravate	–	–	Afanasyeva et al., 2001; Fairweather et al., 2004; Sonderegger et al., 2006; Zafra et al., 2007; Chen et al., 2009; Noutsias et al., 2011; Wu et al., 2016; Chen et al., 2017b
**Cardiac fibrosis**	Aggravate	Aggravate	–	–	Li et al., 2012; Yan et al., 2012; Savvatis et al., 2014; Ye et al., 2020
**Cardiac injury**	Alleviate	–	–	Alleviate	Jia, 2018; Ye et al., 2018b; Ye et al., 2018c
**Heart transplantation**	Aggravate	–	–	–	Wang et al., 2012

Contro, controversial.

## Author Contributions

JY, YW, and ZW wrote this article. LL, ZY, MW, YX, DY, and JZ searched literatures. YL, QJ, and JW provided ideas and financial support.

## Funding

This work was supported by the National Natural Science Foundation of China (No. 81770472 and No. 81560085 to QJ; No. 81760051 to YL).

## Conflict of Interest

The authors declare that the research was conducted in the absence of any commercial or financial relationships that could be construed as a potential conflict of interest.
